# Dynamic Positron Emission Tomography Image Restoration via a Kinetics-Induced Bilateral Filter

**DOI:** 10.1371/journal.pone.0089282

**Published:** 2014-02-27

**Authors:** Zhaoying Bian, Jing Huang, Jianhua Ma, Lijun Lu, Shanzhou Niu, Dong Zeng, Qianjin Feng, Wufan Chen

**Affiliations:** School of Biomedical Engineering, Southern Medical University, Guangzhou, China; CNRS, France

## Abstract

Dynamic positron emission tomography (PET) imaging is a powerful tool that provides useful quantitative information on physiological and biochemical processes. However, low signal-to-noise ratio in short dynamic frames makes accurate kinetic parameter estimation from noisy voxel-wise time activity curves (TAC) a challenging task. To address this problem, several spatial filters have been investigated to reduce the noise of each frame with noticeable gains. These filters include the Gaussian filter, bilateral filter, and wavelet-based filter. These filters usually consider only the local properties of each frame without exploring potential kinetic information from entire frames. Thus, in this work, to improve PET parametric imaging accuracy, we present a kinetics-induced bilateral filter (KIBF) to reduce the noise of dynamic image frames by incorporating the similarity between the voxel-wise TACs using the framework of bilateral filter. The aim of the proposed KIBF algorithm is to reduce the noise in homogeneous areas while preserving the distinct kinetics of regions of interest. Experimental results on digital brain phantom and *in vivo* rat study with typical ^18^F-FDG kinetics have shown that the present KIBF algorithm can achieve notable gains over other existing algorithms in terms of quantitative accuracy measures and visual inspection.

## Introduction

Dynamic positron emission tomography (PET) is a powerful tool that provides useful quantitative information on physiological and biochemical processes [1]. Associative parametric imaging can be achieved by fitting the time activity curves (TACs) at each voxel with a linear or nonlinear kinetic model [2]. However, low signal-to-noise ratio (SNR) in short dynamic frames causes the related noise to be inevitably transferred to the voxel-wise kinetic parameter estimation from the TAC measurements. Thus, PET parametric imaging is essentially an ill-posed problem.

As regards PET parametric imaging, the related reconstructions can be realized by using direct and indirect methods [3]–[5]. Direct reconstruction methods enable accurate compensation of noise propagation from the projection data to the kinetic fitting process by combining kinetic modeling and dynamic image reconstruction into a unitary formula [6]–[9]. Direct reconstruction methods usually require knowledge of the input function, that is, the TAC of tracer concentration in arterial blood. The related data acquisition is known to be invasive and labor intensive, which limits its application in clinical practice. As regards indirect reconstruction methods, dynamic image reconstruction and kinetic analysis are conducted separately. Meanwhile, the image-derived input function method [9]–[13] or reference region method [14], [15] can be employed as an alternative to painful blood sample measurement. To achieve high-quality dynamic PET images, two strategies are commonly used, namely, maximum a posteriori (MAP) image reconstruction and image restoration based post-processing [16]–[20]. MAP image reconstruction with significant noise suppression is performed by incorporating different prior models, such as the spatial quadratic smoothing prior [21], sophisticated edge-preserving priors [17], [22]–[26], anatomical priors [27]–[31], and kinetics-based priors [18], [32], [33]. These MAP approaches, particularly with sophisticated edge-preserving priors, may have limited practical applications in clinic because of implementation complexity and computational burden. As an alternative strategy, image post-processing through spatial filtering has been extensively explored to reduce the noise of individual PET image frames [19], [34]–[37]. A simple and common technique is the use of a Gaussian filter, which performs well in a homogeneous region with evident noise reduction. However, a Gaussian filter usually fails at edges with noticeable spatial resolution loss because of shift invariance. As an extended version of the Gaussian filter, the bilateral filter (BF) was investigated in PET studies with significant gains over the Gaussian filter in terms of noise reduction and resolution preservation [19]. Meanwhile, all these spatial filters should be noted to reduce the noise of individual image frames without considering the kinetic information contained within entire dynamic images. A recently increasing interest in dynamic PET image filtering is the use of the temporal information from dynamic PET data [20], [38]. For example, the information contained in a time-averaged frame was used to filter each individual frame and improve the SNR of desired PET images [38]. In addition, by considering the dynamic PET TAC in each voxel as a vector, Tauber *et al.* designed a spatio-temporal anisotropic diffusion image filter with noticeable gains over the existing methods [20].

In this work, to improve PET parametric imaging accuracy, we present a kinetics-induced bilateral filter (KIBF) to reduce the noise of dynamic image frames by incorporating the similarity between the voxel-wise TACs using the framework of BF [39]. The aim of the present KIBF algorithm is to reduce the noise in homogeneous areas while preserving the distinct kinetics of regions of interest (ROIs). To validate and evaluate the performance of the proposed KIBF algorithm, experiments were conducted through computer simulations and a preclinical rat study with a focus on quantitative accuracy measures and visual inspection.

## Materials and Methods

### Ethics Statement

The rat study was conducted in strict accordance with the recommendations in the Guide for the Care and Use of Laboratory Animals of the National Institutes of Health. The protocol was approved by the Institutional Animal Care and Use Committee of Case Western Reserve University (Permit Number: 2008-0072). All surgery was performed under 2% isoflurane anesthesia, and all efforts were exerted to minimize suffering.

### Brief Review of the BF

The BF was originally proposed by Tomasi and Manduchi for 2D image processing [39]. The discrete version of their BF algorithm can be expressed as follows: Let 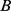 be a discrete grid of voxels and 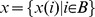 be a noisy image. Given a neighbor window 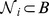 centered at voxel 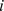, the restored intensity 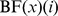 at voxel 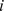 is the weighted average of all intensities of the neighboring voxels 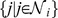 and can be written as
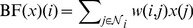
(1)where 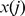 denotes the image intensity at voxel 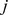, and 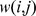 is the weight and consists of a product of two separate filters acting in the spatial and intensity domains. The popular weight 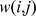 is often defined as Gaussian shape:
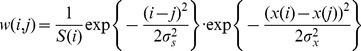
(2)where 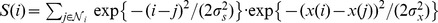 is a normalizing factor to ensure that the weight 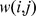 satisfies the conditions of 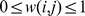 and 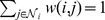. Two parameters 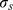 and 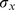 control the geometric proximity and the intensity similarity, respectively.

### Description of the KIBF

Our proposed KIBF adapts the concept of the BF algorithm to make use of both the spatial information and the voxel-wise kinetic information within the entire dynamic PET data. The KIBF algorithm contains two major steps: (a) optimal weight construction using kinetics information; and (b) weighted average using the constructed weights.

#### Optimal Weight Construction

In dynamic PET studies, voxels in physiologically similar regions have similar tissue TAC kinetics. Thus, the TAC tendency can provide the tissue-specific biochemical information for dynamic PET image filtering. Under the framework of the BF algorithm, the weights can be optimally constructed by exploring the voxel-wise kinetic information. In this work, by incorporating the similarity between the voxel-wise TACs, the optimal weights are constructed as follows:
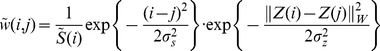
(3)where 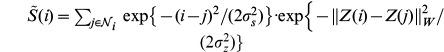 is the normalizing factor. The TAC at voxel 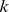 is denoted as 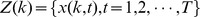, where 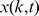 denotes the activity value of voxel 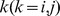 at frame 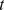, and 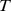 is the total sampling time frames. The similarity measure between two TACs is calculated by the distance measure 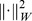, which is defined as 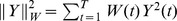, where 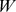 is the vector of weighting factors. An empirical choice of 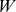 is 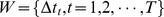, wherein 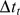 denotes the time duration of the sampling frame 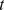. Two factors 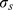 and 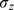 control the spatial voxel neighborhood and the TAC similarity, respectively.

#### Weighted Average

Based on the constructed weights 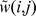, a voxel-wise weighted average operation similar to Equation (1) can be performed on each frame. The present KIBF algorithm can be described as follows:
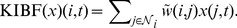
(4)Given that the average weights at voxel 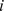 are the same for each frame, the present KIBF algorithm can also be directly performed on the noisy TACs as follows:
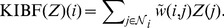
(5)The weighted average of Equation (5) illustrates that the present KIBF algorithm takes advantage of both the spatial and temporal consistencies of the dynamic PET data. As a result, the noise in each individual frame can be remarkably suppressed by the introduction of voxel-wise kinetic information within the entire dynamic frames.

### Parameter Selection for the KIBF Algorithm

For the present KIBF algorithm, three parameters will be determined, namely, the size of the neighbor window 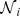 and the controlling parameters 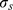 and 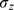. In this study, the minimum mean squared error (MSE) measure and visual inspection of clinical experts were used for parameter selection. The MSE between the original and de-noised dynamic images is defined as 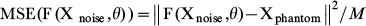, where 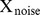 is the noisy dynamic image, 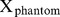 is the original phantom dynamic image, 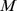 is the total number of voxels, 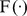 denotes the filtering processing and 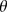 is the filter parameter or parameter set (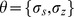 for the present KIBF algorithm) to be determined. Visual inspection was conducted by clinical experts to score from 0 (worst) to 10 (best) for two aspects (namely, noise reduction and edge-preservation) separately for each displayed image set. The higher score indicates the better parameter setting. In the computer simulations, as the ground truth is known, the parameters were all selected by minimizing the MSE with optimized parameter settings. In the preclinical rat study, two clinical experts were asked to score the resultant images in terms of visual inspection on noise reduction and edge-preservation. The resultant images with different parameter settings of the same subject were randomized in order and displayed on the computer screen. The display did not have any indication of which parameter setting was used for the displayed images. Therefore the procedure was completely blind.

#### Selection of Neighbor Window

**Figure 1:**
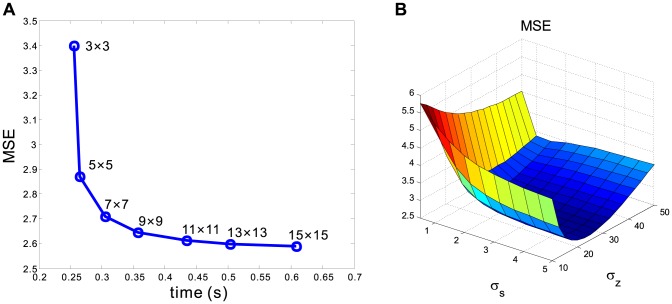
Two MSE plots or parameter selections of the neighbor window (A) and the controlling parameters (B) of KIBF algorithm.

To exploit the neighborhood and similarity information fully for the KIBF algorithm, the neighbor window size 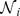 should be sufficiently large. However, the associated computational load will be increased. In this study, extensive experiments with minimum MSE measure and visual inspection from two clinical experts have shown that a 7
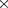
7 neighbor window was adequate for effective noise reduction while retaining computational efficiency. The MSE plot for the selection of the neighbor window size is shown in Figure 1A.

#### Selection of Controlling Parameters

Similar to the BF algorithm, the performance of the KIBF algorithm depends on the selection of two controlling parameters 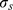 and 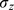. In the implementation, if 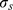 and 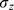 are extremely large, the KIBF filtered images will suffer from smeared edges, whereas the values are extremely small, the desired results cannot achieve noise suppression. Thus, a tradeoff between noise suppression and edge preservation should be achieved by optimizing the two controlling parameters 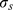 and 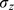 with some reliable image quality measures. In this study, the MSE estimation was used for the computer simulation experiments, and visual inspection by two clinical experts was used for the preclinical rat experiments. Extensive experiments have shown that 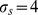 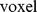 and 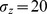 were appropriate for the simulation studies as shown in Figure 1B, and 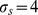 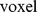 and 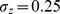 were adequate for the preclinical rat studies.

### Data Acquisitions

#### Computer Simulations

**Figure 2:**
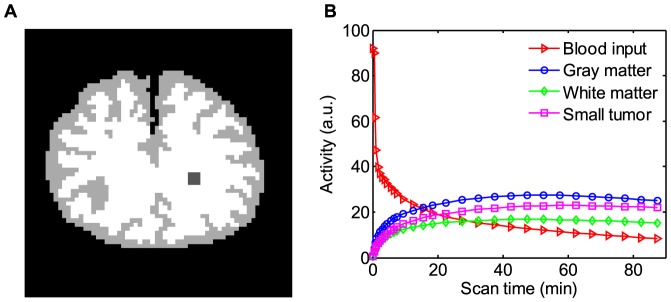
The ^18^F-FDG PET simulation settings. (A) A brain phantom composed of gray matter, white matter and a small tumor; (B) the blood input function and regional time activity curves.

A digital brain phantom [7], [8], as shown in Figure 2A, which consists of gray matter, white matter, and a small tumor within the white matter, was used for the computer simulations. In the simulations, we selected a two-tissue compartment and ^18^F-FDG based tracer kinetic model for glucose metabolism imaging of brain. Based on Feng's model described in [40], the TAC of each region was generated according to the two-tissue compartment model and an analytical blood input function as shown in Figure 2B. The calculation of the kinetic model and the fitting procedure were performed using the COMKAT package (http://comkat.case.edu) [41]. Given the clinical application of the present algorithm, the kinetic parameters 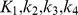 estimated from a set of 70 studies on brain tumor patients described in [42] were used in our simulation and they are listed in Table 1. In this study, the influx rate 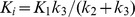, which is related to the glucose metabolic rate by a scaling factor, was analyzed. In addition, the fractional volume of blood in the tissue was set to zero for three target regions. The scanning schedule of dynamic PET data consists of 30 time frames: 4
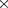
20 s, 4
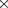
40 s, 4
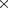
60 s, 4
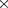
180 s, and 14
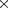
300 s. The TACs were integrated for each frame and forward projected to generate dynamic sinograms and then Poisson noise was added, which resulted in an expected total number of events over 90 min that is equal to 50 million. A filtered back-projection (FBP) method with a ramp filter was used for the dynamic PET reconstruction.

**Table 1:** Kinetic parameters used in the ^18^F-FDG PET simulation.

Regions	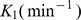	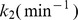	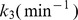	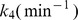
Gray matter	0.1104	0.1910	0.1024	0.0094
White matter	0.0622	0.1248	0.0700	0.0097
Tumor	0.0640	0.0890	0.0738	0.0057

#### Preclinical Dynamic PET Data

The preclinical dynamic PET data were acquired from a female 236 g Sprague-Dawley rat [43]. The rat was injected intravenously with 30.7 MBq of ^18^F-FDG and scanned with a micro-PET R4 system (Siemens Medical Solutions USA, Inc.). The PET scanning schedule was 12
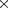
5 s, 12
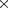
30 s, 5
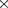
60 s, and 15
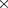
300 s. Corrections for radioactive decay, attenuation, scatter, and dead time were performed before image reconstruction. The dynamic PET images were reconstructed using an image matrix of 128
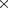
128
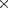
63 with voxel sizes of 0.4922
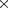
0.4922
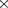
1.220 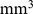 for each frame. An ordered subset expectation maximization (OSEM) algorithm [44] with 16 subsets and 4 iterations was used for all reconstructions. In addition, blood sampling was performed to provide a gold-standard reference, and the input function from the samples was linearly interpolated to construct a final input function. Similar to the computer simulations, the influx rate 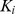 parametric image was calculated based on the final input function.

### Comparison Algorithms

To validate and evaluate the performance of the present KIBF algorithm, the Gaussian filter (GF) and the BF algorithms were adopted for comparison.

#### GF

**Figure 3:**
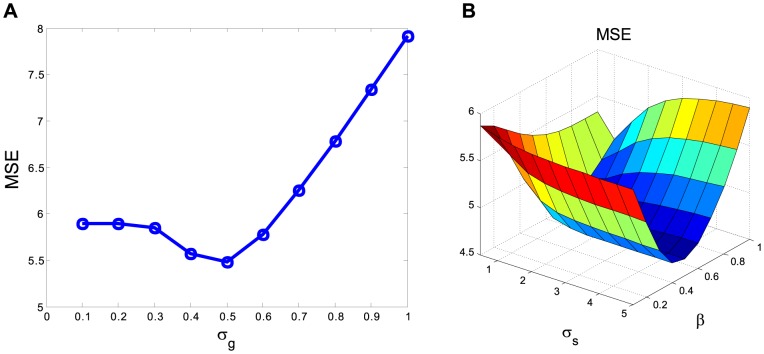
MSE plots for parameter selections of the standard deviation for GF algorithm (A) and the controlling parameters of BF algorithm (B).

The following GF algorithm was implemented for each image frame separately:
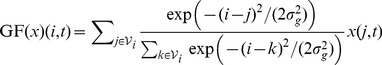
(6)where 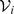 is the neighbor window, and 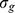 is the standard deviation of the Gaussian function that determines the width of the Gaussian kernel. For each image frame, the parameter 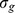 was chosen empirically to yield a minimum MSE as shown in Figure 3A and good visual inspection by two clinical experts.

#### BF

The BF algorithm, as described in Equations (1) and (2), was also applied to each frame separately. Considering the variation of the activity value among different image frames, we proposed a frame-dependent scale parameter form, that is, 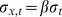, to control the image intensity similarity at frame 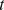, where 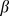 is a scaling factor, and 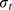 is the estimated noise standard deviation of frame 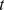. The calculation of 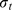 is the same as that used in our previous work [18]. In the implementation, a 7
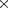
7 neighbor window was used for the BF algorithm. The parameters 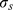 and 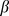 were determined by extensive experiments using minimum MSE estimation, as shown in Figure 3B, and visual inspection by two clinical experts.

## Results

### Phantom Study

#### Comparison of Dynamic PET Activity Images

**Figure 4:**
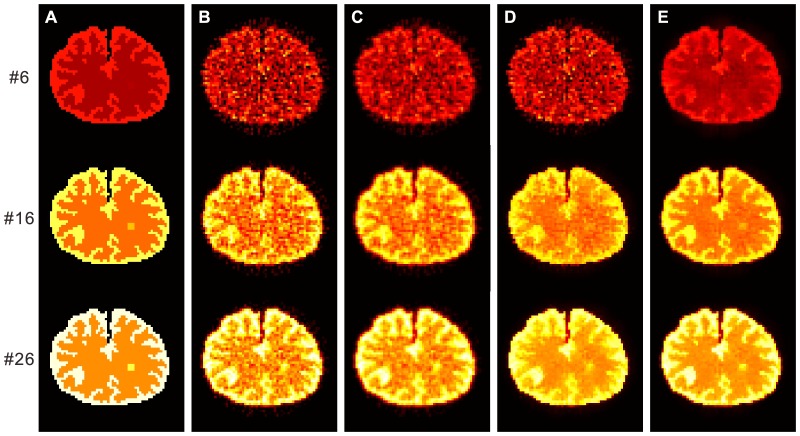
The ground truth and the activity images reconstructed by different algorithms at frames #6, #16, and #26. (A) are the ground truth; (B) are the results from the direct FBP reconstruction; (C) are the results from the FBP images filtered by the GF algorithm (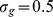 voxel); (D) are the results from the FBP images filtered by the BF algorithm (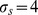 voxel, 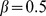); and (E) are the results from the FBP images filtered by the present KIBF algorithm (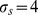 voxel, 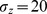). All images are with a same display window.

**Figure 5:**
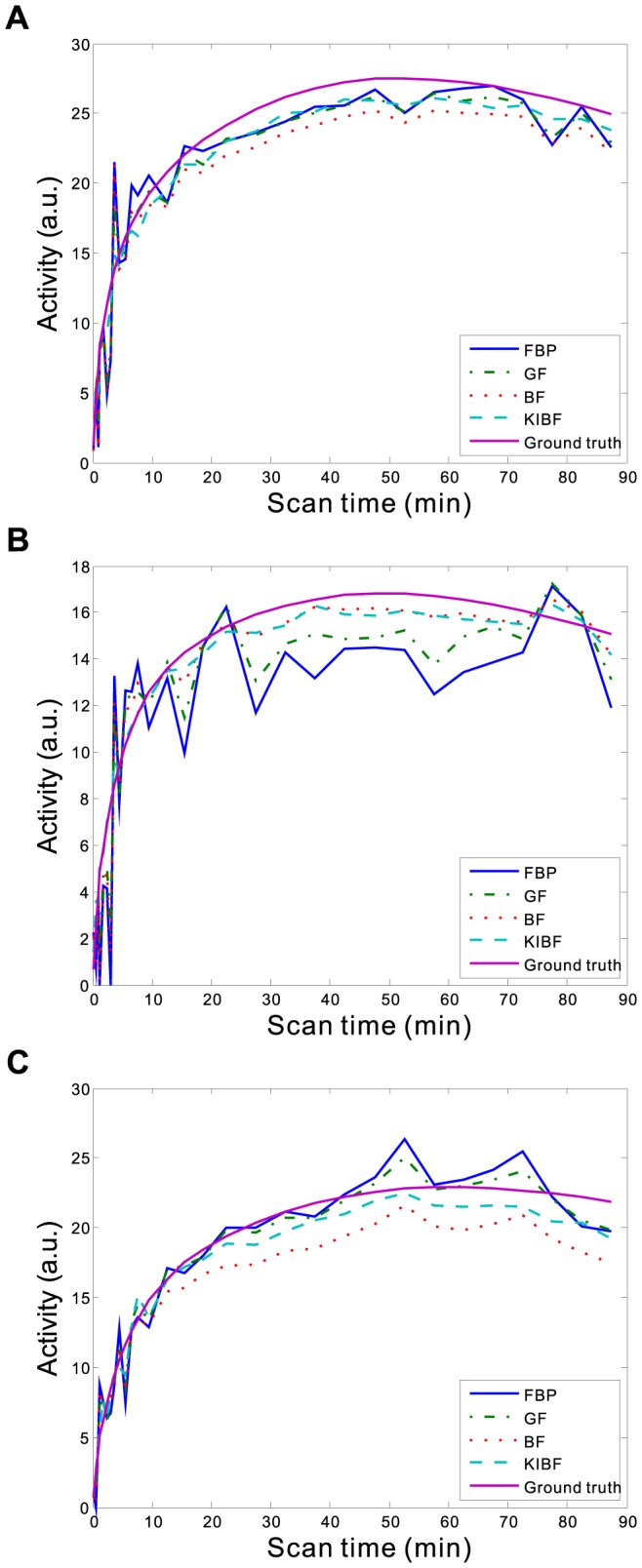
Three TAC plots from different algorithms for the corresponding voxels located in the gray matter (A), white matter (B) and small tumor regions (C), respectively.

**Figure 6:**
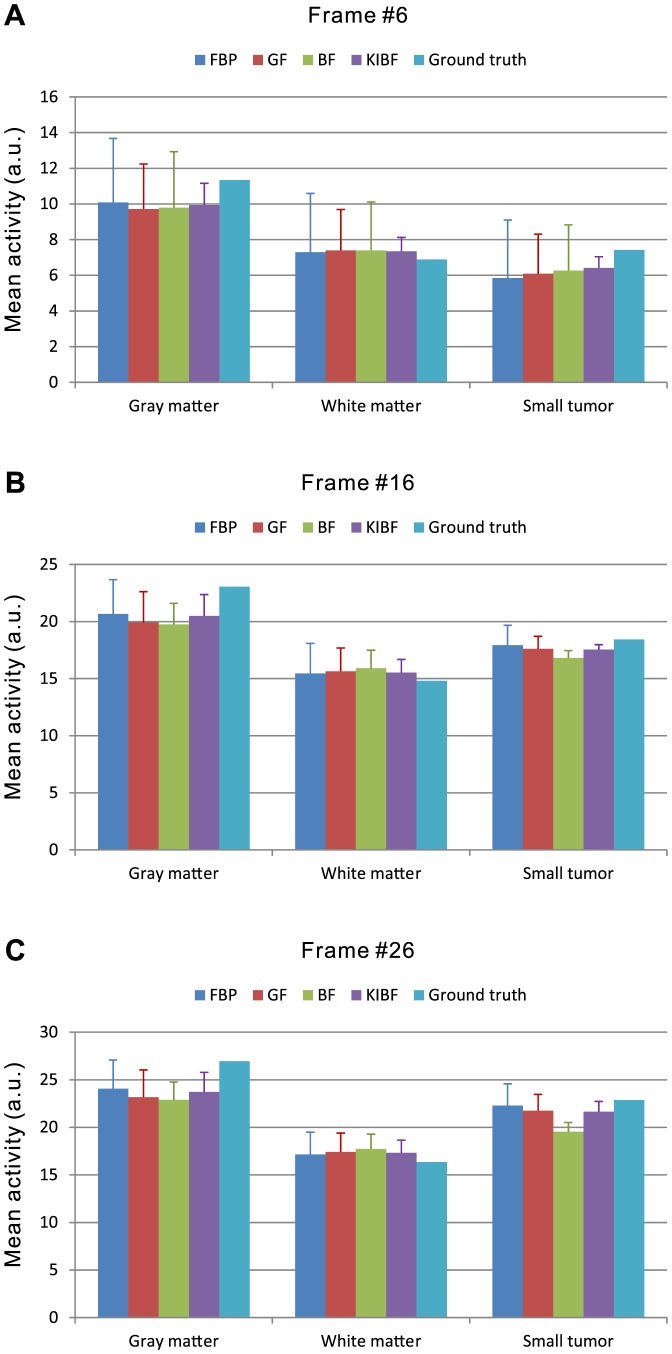
Box plots of the mean activities with standard deviations in the gray matter, white matter and small tumor regions from different algorithms at (A) the frame #6; (B) the frame #16; and (C) the frame #26.

Figure 4 shows the ground truth and the activity images reconstructed by different algorithms at frames #6, #16, and #26. The first column represents the ground truth, the second column shows the results from the direct FBP reconstruction, the third column shows the results from the FBP image filtered by the GF algorithm (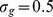 voxel), the fourth column shows the results from the FBP image filtered by the BF algorithm (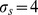 voxel, 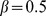); and the fifth column presents the results from the FBP image filtered by the present KIBF algorithm (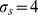 voxel, 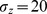). The results demonstrate that the KIBF algorithm can yield significant noise reduction without concealing subtle information compared with other algorithms. To illustrate the effect of temporal information on the smoothing of dynamic frames, we extracted the TACs of three voxels located within the gray matter, the white matter, and the small tumor regions, respectively. Figure 5 shows the TAC plots from different algorithms for the corresponding voxels. We can see that the TACs resulting from the present KIBF algorithm are closer to the ground truth and smoother compared with those of the other three algorithms. Figure 6 shows the box plots of the mean activities with standard deviations in the gray matter, white matter and small tumor regions from different algorithms at frames #6, #16, and #26. We find that the present KIBF algorithm achieves less bias compared with the ground truth as well as less standard deviation than the other algorithms.

**Figure 7:**
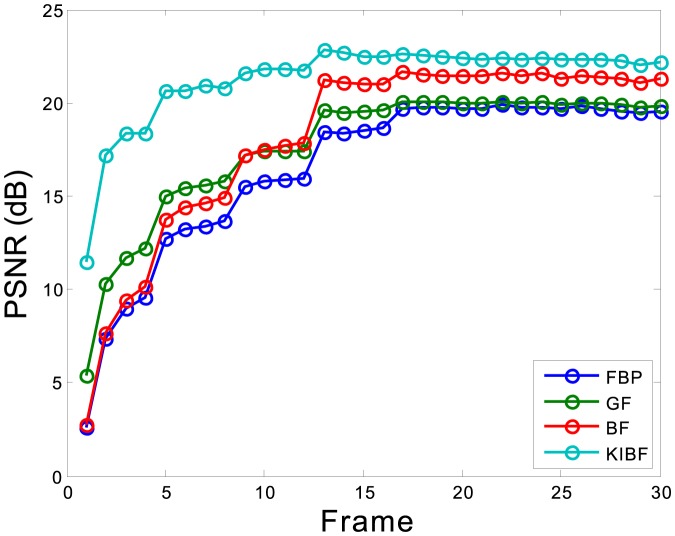
PSNRs at each frame of the activity images reconstructed by different algorithms.

Furthermore, the merits of peak signal-to-noise ratio (PSNR) for each individual frame and the total signal-to-noise ratio (TSNR) for entire dynamic frames were used to measure the bias between the ground truth and estimated values with different algorithms, which is defined as
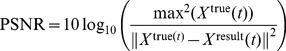
(7)

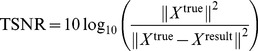
(8)where 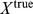 denotes the original phantom image, 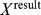 denotes the filtered result, 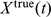 and 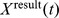 denote the corresponding image at frame 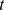, and 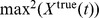 represents the maximum activity value of the original phantom image at frame 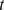. Figure 7 plots the PSNRs at each frame of the activity images reconstructed by the FBP, GF, BF and KIBF algorithms. The PSNR curves illustrate that KIBF has a noticeable gain over the GF and BF algorithms in terms of PSNR measurement, particularly in the early frames. Table 2 lists the TSNRs of the activity images reconstructed by different algorithms. The KIBF algorithm achieves more noticeable gains over other two algorithms in terms of TSNR measurement.

**Table 2:** The TSNRs of the activity images reconstructed by different algorithms.

Methods	FBP	GF	BF	KIBF
TSNRs (dB)	12.92	13.24	13.93	16.29

#### Comparison of PET Parametric Images

**Figure 8:**
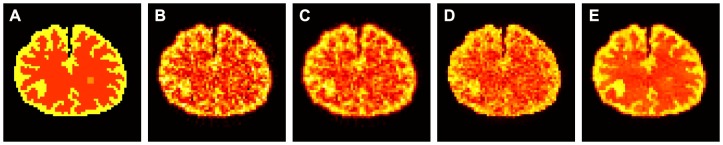
The ground truth and the estimated 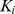 parametric images from the activity images reconstructed by different algorithms. (A) is the ground truth; (B) is the result from the direct FBP reconstruction; (C) is the result from the FBP image filtered by the GF algorithm (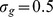 voxel); (D) is the result from the FBP image filtered by the BF algorithm (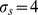 voxel, 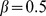); and (E) is the result from the FBP image filtered by the present KIBF algorithm (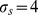 voxel, 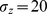). All images are with a same display window.

**Figure 9:**
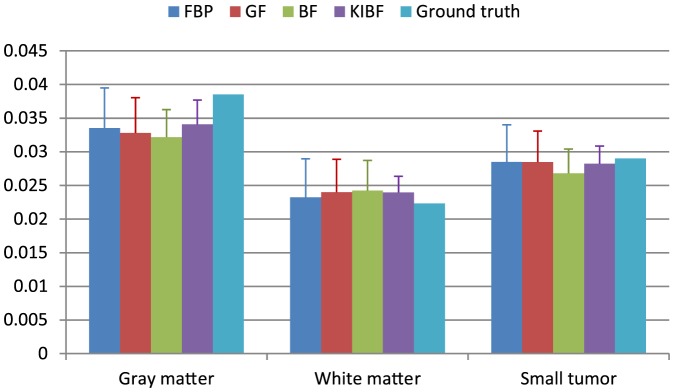
Box plots of the mean value of 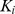 with standard deviations in the gray matter, white matter and small tumor regions from different algorithms.

Figure 8 shows the ground truth and the estimated 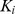 parametric images from the activity images reconstructed by different algorithms: (A) is the ground truth; (B) is the result from the direct FBP reconstruction; (C) is the result from the FBP image filtered by the GF algorithm (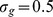 voxel); (D) is the result from the FBP image filtered by the BF algorithm (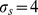 voxel, 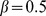); and (E) is the result from the FBP image filtered by the present KIBF algorithm (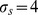 voxel, 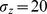). The KIBF algorithm can achieve better performance than other algorithms in terms of both noise reduction and the detailed 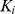 parametric information estimation. Figure 9 represents a description of the mean value of 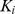 and the standard deviations in the gray matter, white matter, and small tumor regions by different algorithms. We find that the KIBF algorithm achieves less bias compared with the ground truth as well as less standard deviation than the other algorithms.

To evaluate the performance of the KIBF algorithm quantitatively, the regional normalized standard deviation (NSD) versus bias tradeoff curves were studied. Borrowing the definitions in [31], NSD and Bias are written as:
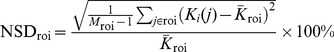
(9)

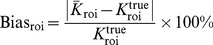
(10)where 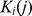 denotes the estimated 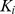 parametric value at voxel 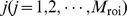 in the region of interest (ROI), 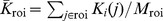 denotes the mean of the estimated 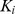 parametric value in the ROI, and 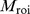 represents the number of voxels in the ROI. For the regional bias (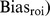, 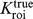 is the known uniform parametric value in the ROI. To quantify NSD versus Bias in the whole brain region for an overall assessment of quantitative performance, 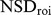 and 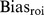 values for the ROIs (gray matter, white matter, and small tumor) were averaged, and weighted based on the size (number of voxels 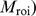 in each ROI.

**Figure 10:**
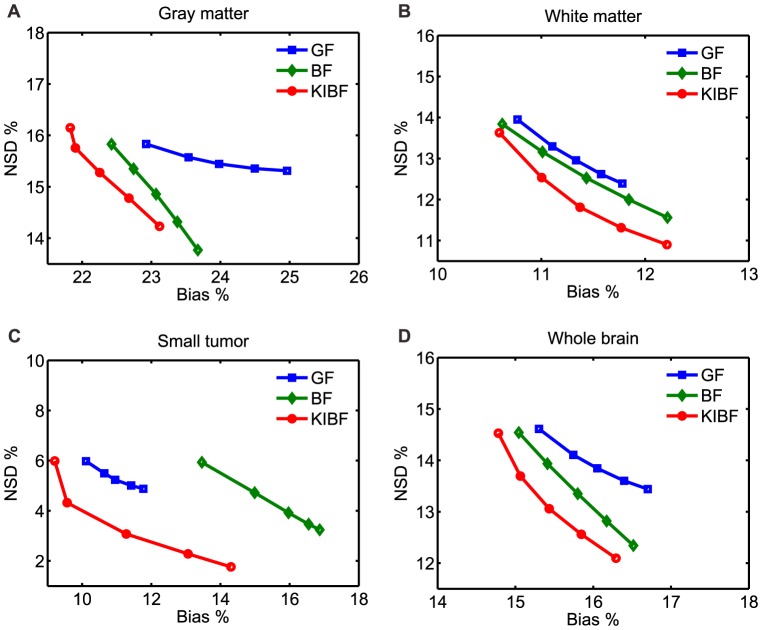
The NSD versus Bias tradeoff curves of the influx rate 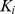 estimated by the GF, BF and KIBF algorithms for different ROIs in the brain phantom.

Figure 10 shows the NSD versus Bias tradeoff curves of the influx rate 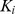 estimated by the GF, BF, and KIBF algorithms for different ROIs in the brain phantom. The parametric images generated from the filtered dynamic activity images with the KIBF algorithm outperform those generated by the other two algorithms based on the NSD versus Bias tradeoff analysis.

### Preclinical Rat Study

**Figure 11:**
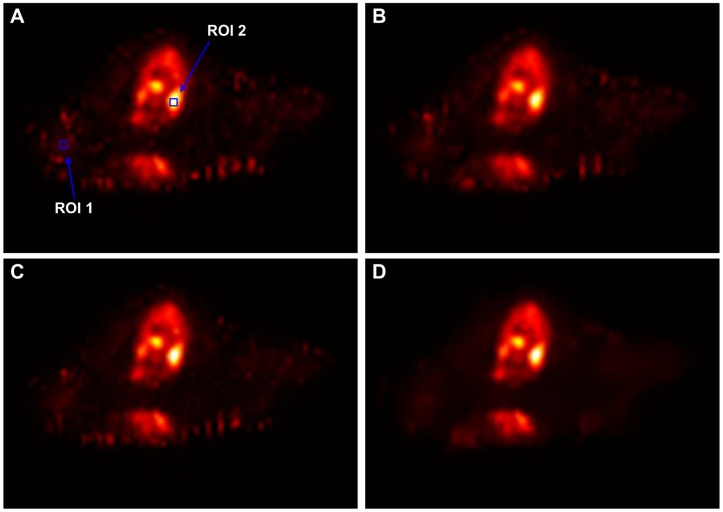
The 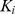 parametric images estimated by different algorithms. (A) is the result from the direct OSEM reconstruction; (B) is the result from the OSEM image filtered by the GF algorithm (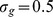 voxel); (C) is the result from the OSEM image filtered by the BF algorithm (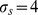 voxel, 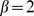); and (D) the result is from the OSEM image filtered by the KIBF algorithm (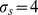 voxel, 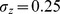). All images are with a same display window.

**Figure 12:**
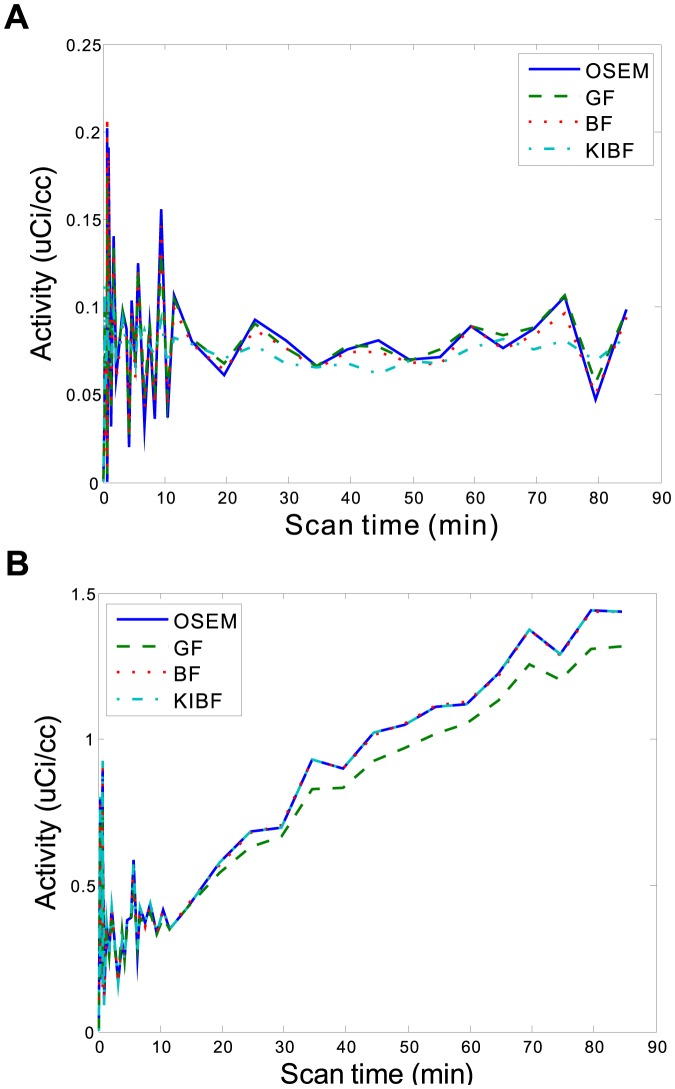
Two TAC plots from different algorithms for the corresponding voxels located in the ROI1 (A) and ROI2 (B), respectively.

Figure 11 shows the transaxial slices of 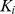 parametric images estimated from: (A) the direct OSEM reconstruction, (B) the OSEM images filtered by the GF algorithm (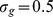 voxel), (C) the OSEM images filtered by the BF algorithm (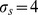 voxel, 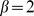), and (D) the OSEM images filtered by the present KIBF algorithm (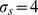 voxel, 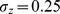). The KIBF algorithm can achieve better performance than other algorithms in terms of both noise reduction and detailed 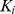 parametric information estimation. Moreover, in the preclinical rat study, we extracted the TACs of two voxels located within a low glucose metabolic region (ROI 1) and a high glucose metabolic region (ROI 2), respectively. The ROIs were indicated by the squares in Figure 11(A). Figure 12 shows the TAC plots from different algorithms for the corresponding voxels. In the low glucose metabolic region, the noise is suppressed strongly by the present KIBF algorithm and the corresponding TAC seems smoother than those resulted from the other algorithms; while in the high metabolic region, the noise is suppressed slightly by the present KIBF algorithm. The results could illustrate that the different smooth strength is dependent on the noise level, and the high glucose metabolic region has a less noise level than the low glucose metabolic region.

## Discussion

To improve PET parametric imaging accuracy, the BF algorithm has been investigated with significant gains over the GF algorithm in terms of noise reduction and resolution preservation [19]. However, as a spatial filter, the BF algorithm merely reduces the noise of individual frames without considering the kinetic information contained in all dynamic images. In this study, we developed the KIBF algorithm to reduce the noise of dynamic images by incorporating the kinetic information using the framework of the BF algorithm. The KIBF algorithm can be regarded as a combination of the spatial domain filter and the temporal TAC filter, as expressed in Equation (3). As a spatio-temporal filter, the KIBF algorithm can reduce the noise in homogeneous areas while preserving the distinct kinetics of ROIs. Moreover, considering the nature of the KIBF calculated from the associative TACs, the algorithm does not require any prior kinetic models typically used in existing approaches [6], [29], [30]. Thus, the KIBF algorithm can be a potential tool to realize accurate dynamic PET imaging. The preliminary results have shown that the KIBF algorithm can achieve better bias-variance properties and quantitative accuracy in generating PET parametric images than the GF and BF algorithms. To validate the present KIBF algorithm, a single rat study is really inadequate. However, more real dynamic PET data are not available in our lab because the dynamic rat PET experiments require very strict experimental conditions. We will do our best to validate the present method using more real dynamic PET data in further research.

Similar to the BF algorithm, a difficult task when performing the KIBF algorithm is parameter selection, including the size of the neighbor window and the controlling parameters. In this study, we used the MSE measure and visual inspection by trial-and-error fashion to optimize the parameters. Meanwhile, the original ground truth is unavailable in practice and more reasonable optimization criteria should be determined according to special application cases. Notably, with the Gaussian noise assumption of PET image [45], [46], Stein's recently developed unbiased risk estimate approach [47]–[50] has demonstrated its capability for parameter selection without requiring the ground truth, which would be helpful for the optimal selection of the parameters of the KIBF algorithm in dynamic PET imaging. This area is an interesting topic for further research.

Another major limitation of the KIBF algorithm is that its performance relies on the alignment between different frames, similar to other time-frames based algorithms [20], [38]. In the implementation, when the TACs associated with voxels located near the interface of different functional regions are a mixture of temporal profiles from the underlying tissues, the KIBF algorithm should be applied by incorporating some motion correction through image registration techniques [51], [52], which also is an interesting research topic.

## Conclusion

In this paper, to achieve accurate kinetic parameter estimation from noisy voxel-wise TACs, we proposed the KIBF algorithm to reduce the noise in homogeneous areas while preserving the distinct kinetics in ROIs. Experimental results on dynamic digital phantom and *in vivo* rat study with typical ^18^F-FDG kinetics have shown that the KIBF algorithm can achieve noticeable gains over other existing algorithms in terms of quantitative accuracy measures and visual inspection. In the future work, we plan to explore more reasonable methodology for parameter selection and evaluate the KIBF algorithm in clinical human dynamic PET studies.
